# Validation of the Dynamic Imaging Grade of Swallowing Toxicity for Amyotrophic Lateral Sclerosis

**DOI:** 10.1111/nmo.70008

**Published:** 2025-03-03

**Authors:** Veena Kallambettu, Justine Dallal York, Terrie Vasilopolous, Katherine Hutcheson, Emily Plowman

**Affiliations:** ^1^ Aerodigestive Research Core Laboratory The Ohio State University Columbus Ohio USA; ^2^ Department of Otolaryngology—Head & Neck Surgery The Ohio State University Columbus Ohio USA; ^3^ Department of Anesthesiology and Orthopaedic Surgery & Sports Medicine University of Florida Gainesville Florida USA; ^4^ Department of Head & Neck Surgery The University of Texas MD Anderson Cancer Center Houston Texas USA

**Keywords:** ALS, DIGEST, dysphagia

## Abstract

**Introduction:**

Although dysphagia is prevalent in persons with amyotrophic lateral sclerosis (pALS) and is associated with morbidity and mortality, no validated outcomes currently exist for the gold standard videofluoroscopy (VF) exam. We therefore sought to psychometrically validate the Dynamic Imaging Grade of Swallowing Toxicity (DIGEST) scale in pALS.

**Methods:**

One hundred pALS attended a research evaluation and underwent a standardized VF and validated clinical outcomes of oral intake (FOIS), perceived swallowing impairment (EAT‐10), and ALS disease progression (ALSFRS‐Revised). Duplicate, independent, and blinded VF ratings were completed using the DIGEST and MBSImP scales. Weighted kappa, ANOVAs (Tukey's HSD, Welch's correction), and Chi‐square analyses were performed to determine intra‐ and inter‐rater reliability, criterion validity, and construct validity of the DIGEST scale for use in pALS.

**Results:**

The mean age was 64.4(SD = 10.4), 50% were male, and the average ALS duration was 28.2 months (SD = 22.2). Excellent intra‐rater (kappa = 0.92–1.0) and inter‐rater (kappa = 0.94) reliability were noted for DIGEST ratings. DIGEST grades significantly discriminated pharyngeal pathophysiology (MBSImP, *F*(3,96) = 24.7, *p* < 0.0001), perceived dysphagia (EAT‐10, *F*(3,40) = 20.8, *p* < 0.0001), oral intake (FOIS, *X* ^2^:25.4, df = 3, *p* < 0.0001), ALS bulbar disease progression (ALSFRS‐bulbar, *F*(3,93) = 20.8, *p* < 0.0001) with main effects noted for all analyses. Post hoc pairwise comparisons noted differences across all DIGEST grades with the exception of DIGEST 2 versus 3 (moderate vs. severe dysphagia), *p* > 0.05.

**Conclusions:**

These data confirm that the DIGEST scale is a reliable and valid VF outcome for use in pALS to distinguish normal versus impaired swallowing and mild versus moderate or severe dysphagia for use in clinical practice and as a clinical trial endpoint marker.

## Graphical abstract

This study sought to psychometrically validate the Dynamic Imaging Grade of Swallowing Toxicity (DIGEST) scale in persons with amyotrophic lateral sclerosis (pALS). Data confirm that the DIGEST scale is a reliable and valid VF outcome tool for use in clinical practice and as a clinical trial endpoint marker in pALS.

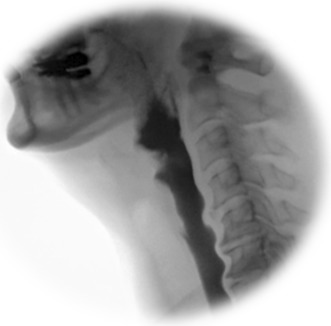

**Summary**

- DIGEST scale is valid video fluoroscopic outcome tool for use in Amyotrophic Lateral Sclerosis (ALS).
- It serves to distinguish normal versus impaired swallowing for use in clinical practice and as a clinical trial endpoint marker.

## 1 Introduction

Dysphagia is reported in up to 85% of persons with amyotrophic lateral sclerosis (pALS) and is associated with significant morbidity and mortality [1, 2] warranting early detection and accurate monitoring of swallowing impairment throughout ALS disease progression [1, 3]. In a large dataset of pALS, Robison and colleagues (2022) reported an overall prevalence in swallowing efficiency impairments of 73% and swallowing safety impairments of 48%. They also noted that impairments in efficiency were four times more likely to represent the initial functional impairment [4].

Videofluoroscopy (VF) is widely recognized as the “gold‐standard” dysphagia exam that provides direct visualization of bolus flow from the oral cavity through the pharynx and into the esophagus to appraise swallowing safety (ability to protect the airway of bolus invasion) and efficiency (how well a food or liquid clears through the pharynx) [5, 6, 7, 8, 9]. Indeed, VF was recommended in the recent Northeastern ALS (NEALS) bulbar best practice guidelines to assess and monitor dysphagia in pALS [1]. However, to date, no validated VF rating scale exists to implement in clinical practice or research settings in pALS. While rating scales continue to be developed, one of the most critical aspects of scale validation is for them to be population specific [10, 11]. In ALS, the progression of disease and onset can have unique implications for dysphagia presentation [12]. With known variability in pathophysiology and associated functional outcomes, scales validated for a different population may not be sensitive enough to account for the unique implications in pALS. Although the penetration‐aspiration scale [13] is a widely used validated scale, it only measures one aspect of dysphagia (i.e., safety) which, given the dysphagia manifestation of functional swallowing impairment profiles noted above in pALS [4] does not adequately capture swallowing impairment in this patient population.

Recently, the *D*ynamic *I*maging *G*rade of *S*wallowing *T*oxicity (DIGEST) scale was developed and validated as a functional rating scale indexing both swallowing safety and efficiency in individuals with head and neck cancer (HNC) [14, 15] that aligns with the National Cancer Institute's Common Terminology Criteria for Adverse Events (CTCAE). Given that most validated clinical scales measure a singular functional aspect of swallowing (i.e., safety), the global functional nature of the DIGEST scale represents a significant advancement in dysphagia measurement and diagnostics. The DIGEST utilizes a subjective visual‐perceptual rating of the perceived percentage of bolus residue in the pharynx at a defined time point (after the initial swallow) using a 4‐point ordinal scale. Safety ratings are derived from penetration‐aspiration scale (PAS) scores [16] and modifiers related to both frequency and amount of bolus airway invasion. DIGEST efficiency and safety grades are derived, and a total DIGEST grade integrates the safety and efficiency results to derive an overall rating of swallowing dysfunction. The DIGEST method does not require specialized software or extensive training to proficiently perform and is relatively quick, making it a desirable and clinically feasible outcome for use in real‐world clinical settings [17].

Given the paucity of ALS disease‐specific VF‐validated outcomes, dysphagia manifestation profiles in pALS, and the nature of the DIGEST outcome, we sought to evaluate the *reliability* (intra‐ and inter‐rater), *criterion validity*, and *construct validity* of the DIGEST scale for use in pALS.

## 2 Methods

### 2.1 Participants

One hundred individuals who were enrolled in a natural history longitudinal study (NCT02962050) were included in this study. Inclusion criteria were as follows: (1) diagnosis of ALS per the El Escorial criteria [18], (2) no documented allergies to barium, (3) not pregnant, and (4) still consuming some form of oral intake. This study was approved by the institutional review board, and all participants signed an informed consent to participate (IRB201602098). Data from the one testing session were used for this specific analysis. One hundred VF exams performed in pALS were retrieved from the lab’s database, with 25 VF studies randomly selected for total DIGEST grade severities corresponding to functional (0), mild (1), and severe (3) and 24 VF studies for moderate (2). This dataset only included one exam with a corresponding DIGEST score of 4 (i.e., life‐threatening dysphagia) that was included.

### 2.2 Procedures

All participants underwent a standardized videofluoroscopy (VF), the functional oral intake scale (FOIS) [19], completed the Eating Assessment Tool‐10 (EAT‐10) [20], and the ALS Functional Rating Scale–Revised [21].

*Standardized VF*: VF swallow studies were conducted in the laboratory where participants sat upright in a Trans‐Motion Medical TMM3 Videofluoroscopy Swallow Study Treatment Chair (Ocala, FL). Images were captured continuously in the lateral plane using a GE Healthcare fluoroscopic mobile C‐Arm unit (OEC 9900 Elite) and recorded at 30 frames per second (FPS) using a TIMS DICOM Audiovisual recording system (Version 3.2, TIMS Medical, TM, Chelmsford, MA). A standardized bolus protocol was utilized using 40% weight/volume with Varibar barium sulfate products (Bracco Imaging, Monroe Township, NJ) that have been mapped to the International Dysphagia Diet Standardization Initiative (IDDSI) levels [22]. The standardized bolus protocol was: three cued swallows of 5 cc thin liquid boluses from a 30 cc medicine cup (IDDSI level 0), one un‐cued comfortable cup sip of thin liquid from a cup filled to 90 cc (IDDSI level 0), three cued swallows of 5 cc moderately thick boluses by spoon (IDDSI level 3), two cued swallows of 5 cc pudding trials (IDDSI level ≥ 4) by spoon and one cued swallow of ¼ graham cracker coated with pudding (IDDSI level 7). The bailout criterion for VFs used to maintain participant safety was three episodes of gross aspiration (> 25% of bolus volume swallowed) [1, 2] and/or > 75% pharyngeal residue remaining despite attempts to clear it.

*Criterion Validity*—Pharyngeal Swallowing: Modified Barium Swallow Impairment Profile (MBSImP) is a standardized physiologically anchored rating scale for assessment of swallowing that comprises 17 distinct components(lip closure, tongue control, bolus preparation/mastication, bolus transport/lingual motion, oral residue, initiation of the pharyngeal swallow, soft palate elevation, laryngeal elevation, anterior hyoid excursion, epiglottic movement, laryngeal vestibular closure, pharyngeal stripping wave, pharyngeal contraction, pharyngoesophageal segment opening, tongue base retraction, pharyngeal residue, and esophageal clearance) that are scored on a 3‐ or 4‐point ordinal scale and grouped into either the oral impairment domain (6 components), pharyngeal impairment domain (10 components), or the esophageal impairment domain (1 component). Similar to the original DIGEST validation study [14] the MBSImP pharyngeal impairment score served as the reference criterion to assess the criterion validity of pharyngeal swallowing.

*Criterion Validity—Oral Intake*: The Functional Oral Intake Scale (FOIS) [19] is a validated 7‐point ordinal clinician rating scale of oral intake and was used to serve as the criterion reference standard for oral intake in this study.

*Criterion Validity—Patient‐reported swallowing impairment*: The EAT‐10 is a validated patient‐reported outcome (PRO) of perceived swallowing impairment [20]. It contains 10 items a patient rates using a 5‐point ordinal scale with overall scores ranging from 0 (no perceived difficulty with swallowing) to 40 (severe perceived difficulty with swallowing). The EAT‐10 has been reported to distinguish safe versus unsafe swallowing in pALS [23, 24] and pALS with and without dysphagia [23, 24].

*Construct Validity—ALS Functional Rating Scale–Revised*: The ALSFRS–Revised bulbar subscale [21] comprises three items related to swallowing, saliva, and speech that patient rates on a 5‐point ordinal scale. Subscale scores range from 0 (severe bulbar dysfunction) to 12 (no perceived bulbar dysfunction).

*DIGEST ratings*: VF studies were analyzed by six trained raters who performed independent and blinded DIGEST ratings following standardized published procedures [15]. Each VF study was randomly assigned to two raters, with a complete agreement (100%) of DIGEST grades required. Following the completion of all ratings, a consensus meeting was held with all six raters in attendance to resolve any inconsistent ratings. For the purposes of intra‐rater reliability, 20% of VF studies (*n* = 20) were evaluated twice by the same rater in a blinded fashion 1 month following the submission of their initial ratings. VF studies were assigned randomly across raters, with each rater being assigned 33 or 34 unique VF studies and six duplicate VF studies (i.e., total of 39 or 40 VF studies).

### 2.3 Statistical Analysis

Weighted kappa analysis was conducted to assess inter‐ and intra‐rater agreement among multiple raters of DIGEST ratings. ANOVA (Analysis of Variance) with Tukey's HSD (Honestly Significant Difference) and Welch's correction was performed to compare means between DIGEST scores and established standards for pharyngeal dysphagia (MBSImP‐pharyngeal domain; EAT‐10) and ALSFRS‐R. Chi‐square test was carried out to examine the association between DIGEST and FOIS. Across all analyses, *p* < 0.05 was considered statistically significant. All statistical analyses were completed using JMP Pro version 13.0 (SAS Institute Inc., Cary, North Carolina).

## 3 Results

### 3.1 Participant Characteristics

Participant mean age was 64.4 years (SD: 10.4) of whom an equal number were male and female (*n* = 50 male, *n* = 50). Average ALS disease duration was 28.2 months (SD: 22.2), mean ALSFRS‐R score was 34.3 (SD: 7.9), and disease onset type was spinal (*N* = 39 (39%)), bulbar (*N* = 54 (54%)), and mixed (*N* = 7 (7%)).

### 3.2 DIGEST Grade Distribution

**FIGURE 1:**
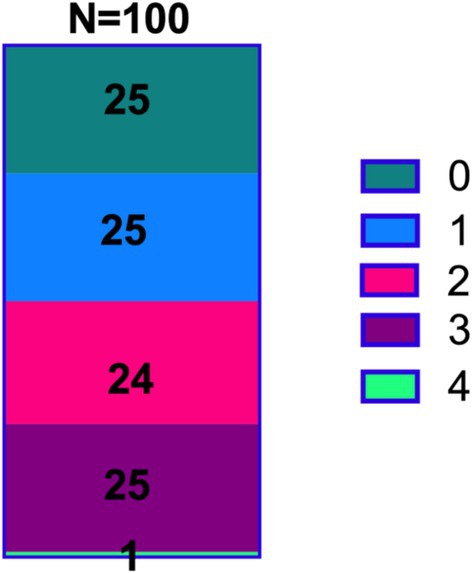
Distribution of Dynamic Imaging Grade of Swallowing Toxicity total grades.

DIGEST subscale (efficiency and safety) and total grades are summarized in Table 1 and reveal a relatively equal distribution of ratings in the functional (0), mild (1), moderate (2), and severe (3) severity DIGEST total grades; however, with only one case of life‐threatening (4) total DIGEST grade. Figure 1 provides a graphical representation.

**TABLE 1:** Distribution of Dynamic Imaging Grade of Swallowing Toxicity—efficiency, safety, and total grades.

	Efficiency grade	Safety grade	Total grade
0 (Functional)	36 (36%)	44 (44%)	25 (25%)
1 (Mild)	41 (41%)	17 (17%)	25 (25%)
2 (Moderate)	3 (3%)	19 (19%)	24 (24%)
3 (Severe)	19 (19%)	20 (20%)	25 (25%)
4 (Life‐threatening)	1 (1%)	0 (0%)	1 (1%)

Safety and efficiency subscale grades demonstrated greater variability across DIGEST grades, with a notable skew of data points in the lower grades, particularly for DIGEST efficiency grades.

For statistical analysis, given that only one participant presented with a DIGEST rating of 4 (life‐threatening), a score of “4” was grouped with “3” (severe).

### 3.3 Reliability

Excellent intra‐rater reliability was revealed with weighted kappa scores ranging between 0.92 and 1.00 across the six raters for total DIGEST grades. Similarly, inter‐rater reliability was excellent for total DIGEST grades (k_w_ = 0.85).

### 3.4 Criterion Validity

**FIGURE 2:**
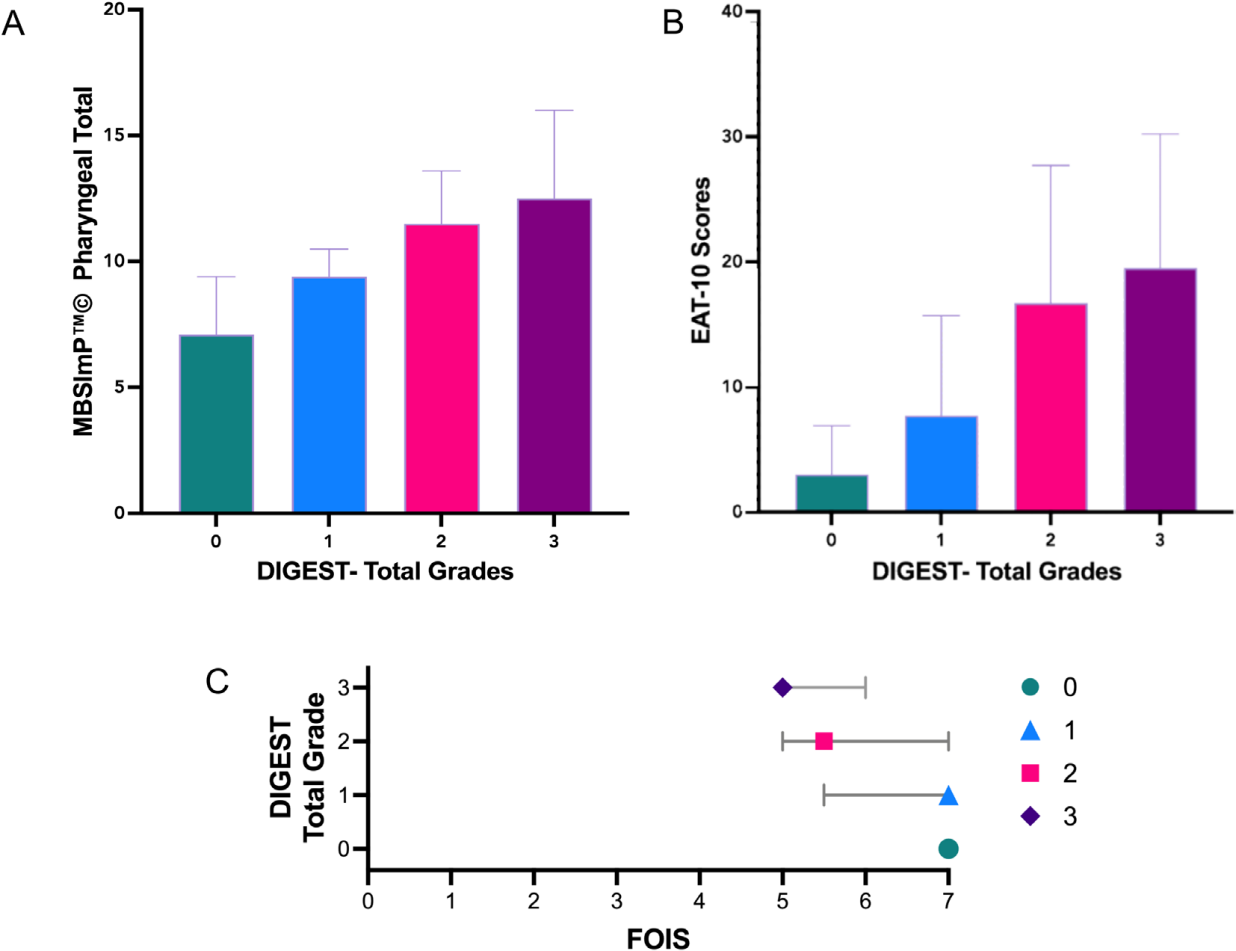
Criterion validity of the Modified Barium Swallow Impairment Profile (A), Eating Assessment Tool‐10 (B) summary scores, and Functional Oral Intake Scale (C) mean and interquartile range scores across Dynamic Imaging Grade of Swallowing Toxicity Total grades. Abbreviations: DIGEST, Dynamic Imaging Grade of Swallowing Toxicity; EAT‐10, Eating Assessment Tool‐10; FOIS, Functional Oral Intake Scale; IQR, Interquartile range; MBSImP, Modified Barium Swallow Impairment Profile.

Criterion validity of the DIGEST scale was appraised against the Modified Barium Swallow Impairment Profile (MBSImP‐Pharyngeal Domain) [25], FOIS [19], and EAT‐10 [20] validated scales of swallowing, oral intake, and patient report, respectively. Figure 2 provides a graphical representation of criterion validity.

- *Pharyngeal Swallowing*: MBSImP‐Pharyngeal Domain scores were noted to significantly differ by total DIGEST grades, with a main effect observed [*F*(3,96) = 24.7, *p* < 0.0001]. Post hoc analysis revealed significant between‐group differences for each DIGEST grade pairwise comparison except for between DIGEST grades 2 and 3 (i.e., moderate vs. severe). MBSImP summary data across DIGEST grades and post hoc pairwise comparisons are displayed in Table 2.
- *Oral intake*: FOIS scores across DIGEST grades are summarized in Table 3. *Overall*, FOIS scores were noted to differ across DIGEST grades, X_2_ (4) = 27.88, *p* < 0.0001.
- *Patient Report*: EAT‐10 total DIGEST scores are summarized in Table 4. EAT‐10 scores differed by DIGEST grade, *F*(3,40) = 20.9, *p* < 0.0001. Post hoc analyses revealed significant differences between all DIGEST grade pairwise comparisons except for between DIGEST grades 2 versus 3 (moderate vs. severe) and grades 0 versus 1 (functional vs. mild).

**TABLE 2:** Modified Barium Swallow Impairment Profile summary data across Dynamic Imaging Grade of Swallowing Toxicity severity grades.

DIGEST total grade	Mean (SD)	Median (IQR)	DIGEST pairwise comparisons, significant differences*
0 (Functional)	7.1 (2.3)	7 (5.5, 9)	0 vs. 1, *p* = 0.0049* 0 vs. 2, *p* < 0.0001* 0 vs. 3–4, *p* < 0.0001*
1 (Mild)	9.4 (1.1)	10 (9, 10)	1 vs. 2, *p* = 0.0167* 1 vs. 3–4, *p* = 0.0001*
2 (Moderate)	11.5 (2.1)	11 (10, 13)	2 vs. 3–4, *p* = 0.5079
3 (Severe)	12.5 (3.5)	12.5 (10, 15)	—

Abbreviations: DIGEST, Dynamic Imaging Grade of Swallowing Toxicity; IQR, interquartile range; SD, standard deviation.

* indicates a significant pairwise difference.

**TABLE 3:** Functional Oral Intake Scale summary data across Dynamic Imaging Grade of Swallowing Toxicity severity grades.

DIGEST total grade	Mean (SD)	Median (IQR)	DIGEST pairwise comparisons, significant differences*
0 (Functional)	6.7 (0.6)	7 (7, 7)	0 vs. 1, *p* = 0.5546 0 vs. 2, *p* = 0.0254* 0 vs 3‐4, *p* < 0.0001*
1 (Mild)	6.3 (1.1)	7 (5.5, 7)	1 vs 2, *p* = 0.4006 1 vs 3‐4, *p* = 0.0013*
2 (Moderate)	5.8 (0.9)	5.5 (5, 7)	2 vs 3‐4, *p* = 0.1248
3 (Severe)	5.0 (1.8)	5 (5, 6)	—

Abbreviations: DIGEST, Dynamic Imaging Grade of Swallowing Toxicity; IQR, interquartile range; SD, standard deviation.

* indicates a significant pairwise difference.

**TABLE 4:** Eating Assessment Tool‐10 summary data across Dynamic Imaging Grade of Swallowing Toxicity grades.

DIGEST total	Mean (SD)	Median (IQR)	DIGEST pairwise comparisons, significant differences*
0 (Functional)	3.0 (3.9)	0 (0,5.5)	0 vs. 1, *p* = 0.2463 0 vs. 2, *p* < 0.0001* 0 vs. 3–4, *p* < 0.0001*
1 (Mild)	7.7 (8.0)	6 (1.5, 12)	1 vs. 2, *p* = 0.0030* 1 vs. 3–4, *p* < 0.0001*
2 (Moderate)	16.7 (11.0)	16.5 (5.5, 27.75)	2 vs. 3–4, *p* = 0.6818
3 (Severe)	19.52 (10.7)	20 (11, 28)	—

Abbreviations: DIGEST, Dynamic Imaging Grade of Swallowing Toxicity; IQR, interquartile range; SD, standard deviation.

* indicates a significant pairwise difference.

### 3.5 Construct Validity

**FIGURE 3:**
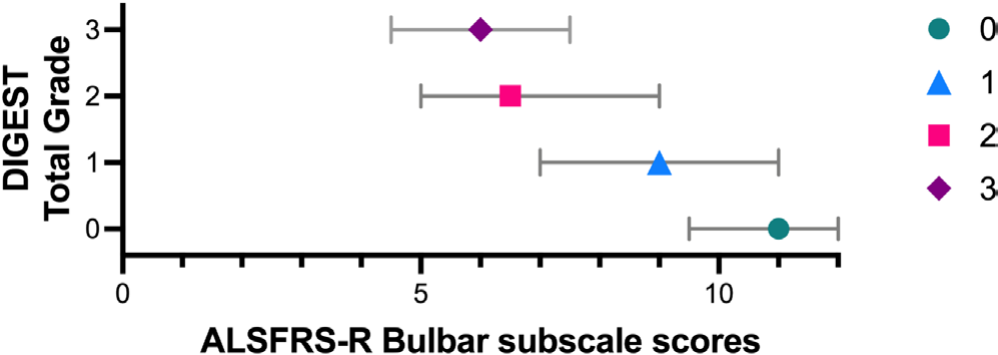
Amyotrophic Lateral Sclerosis Functional Rating Scale–Revised bulbar subscale median and interquartile range scores across Dynamic Imaging Grade of Swallowing Toxicity total grades. Abbreviations: ALSFRS‐R, Amyotrophic Lateral Sclerosis Functional Rating Scale–Revised, DIGEST, Dynamic Imaging Grade of Swallowing Toxicity; IQR, Interquartile range.

ALSFRS‐R bulbar subscale scores were noted to differ by DIGEST grades *F*(3,93) = 20.8, *p* < 0.0001. Post hoc analyses revealed significant differences for each DIGEST grade pairwise comparison except for between DIGEST grades 2 and 3 (i.e., moderate vs. severe dysphagia). ALSFRS‐R bulbar subscale summary data across DIGEST grades and post hoc pairwise comparisons are displayed in Table 5 and graphically represented in Figure 3.

**TABLE 5:** Amyotrophic Lateral Sclerosis Functional Rating Scale–Revised bulbar subscale summary data across Dynamic Imaging Grade of Swallowing Toxicity grades.

DIGEST total	Mean (SD)	Median (IQR)	DIGEST pairwise comparisons, significant differences*
0 (Functional)	10.5 (1.8)	11 (9.5, 12)	0 vs. 1, *p* = 0.0431* 0 vs. 2, *p* < 0.0001* 0 vs. 3–4, *p* < 0.0001*
1 (Mild)	8.8 (2.5)	9 (7, 11)	1 vs. 2, *p* = 0.0229* 1 vs. 3–4, *p* < 0.0001*
2 (Moderate)	6.9 (2.5)	6.5 (5, 9)	2 vs. 3–4, *p* = 0.3921
3 (Severe)	5.9 (2.1)	6 (4.5, 7.5)	—

Abbreviations: DIGEST, Dynamic Imaging Grade of Swallowing Toxicity; IQR, interquartile range; SD, standard deviation.

* indicates a significant pairwise difference.

## 4 Discussion

DIGEST is a recently validated VF outcome of pharyngeal swallowing dysfunction for use in individuals with HNC. In this population, the DIGEST has filled an unmet need in dysphagia diagnostics to offer a psychometrically robust, yet pragmatic, quick evidence‐based practice tool giving clinicians a reproducible and common language for routine dysphagia imaging studies. Unfortunately, no such validated scale for the measurement of pharyngeal swallowing dysfunction exists for pALS, motivating the current study that sought to psychometrically validate the DIGEST outcome for use in pALS. Results indicated that the DIGEST scale demonstrated excellent reliability, criterion validity, and construct validity to grade pharyngeal swallowing impairment in pALS.

The observed excellent intra‐rater reliability is concordant with the original DIGEST validation report in cancer to indicate a high level of confidence in the reproducibility of DIGEST ratings and internal calibration of raters for the use of this tool in both clinical and research settings. The noted high reliability supports the utility of the DIGEST scale for use in both clinical and research trials to monitor disease progression over time or to benchmark treatment effects across time points in a given patient. The current intra‐ and inter‐rater reliability results are slightly higher than the original DIGEST validation studies in the cancer population for both VF [14, 15] and fiberoptic endoscopic instrumental imaging modalities of swallowing [26] This likely reflects the calibration occurring in the consensus rating process.

In this validation study of the DIGEST in pALS, criterion validity was determined by examining DIGEST grades against established outcomes of swallowing physiology (MBSImP), oral intake (FOIS), and patient report (EAT‐10). Results in this cohort of pALS supported the criterion validity of the DIGEST in pALS with the three pillars of pharyngeal swallowing assessment. Our findings confirmed the alignment of DIGEST severity grades with the well‐established MBSImP, with increasing pharyngeal impairment scores with increasing DIGEST grade and a more than doubling in the MBSImP pharyngeal impairment score between DIGEST grade 0 and 4 (7 vs. 17). Significant between‐group differences were noted for every DIGEST grade comparison except for between DIGEST grades 2 and 3 (mild vs. moderate impairment severity grades). Similar MBSImP distributions among DIGEST grade 2 (moderate) and 3 (severe) may reflect similar physiologic profiles underlying these higher‐grade dysphagia states. DIGEST grades were aligned with oral intake in this cohort of 100 pALS, with lower median FOIS scores observed with increasing DIGEST grades (i.e., increasing swallowing impairment), specifically ranging from 7 (full oral diet) for DIGEST grade 0 (functional) to a FOIS score of 1 (no oral intake of any kind) for the individual with a DIGEST grade of 4 (life‐threatening dysphagia). A significant difference in FOIS scores was noted between individuals with a functional DIGEST grade (0) versus those with moderate–severe swallowing impairment grades (i.e., DIGEST grades > 2); however, not for pALS with mild swallowing impairment scores (i.e., DIGEST grade 1). This finding likely reflects the clinical reality where a mild swallowing deficit does not necessarily result in a drastic change in oral intake status. Many pALS may naturally employ simple compensatory strategies (e.g., double swallow, liquid wash) to continue oral intake of preferred food textures, and a clinical change in food texture avoidance or modification is only evident when the change in severity is more pronounced. Similar to MBSImP results, FOIS also did not differ between pALS with DIGEST mild versus moderate impairment severity grades (i.e., grades 2 and 3) with median scores of 5.5 and 5.0, respectively. As noted above, oral intake and feeding status may not follow an exactly linear dose–response outcome. FOIS levels 5 and 6 are both defined as total oral diet, with FOIS level 5 described as requiring special preparation or compensations and FOIS level 6 not requiring special preparation but avoiding certain foods. Overall, however, the DIGEST aligned with oral intake consumption and distinguished FOIS scores between pALS with normal swallowing—mild/early impairment and those with moderate to severe dysphagia.

Patient‐perceived swallowing impairment was also related to DIGEST grades in this group of pALS, with increasing (worse) EAT‐10 scores observed with increased DIGEST grades. Indeed, mean EAT‐10 scores more than doubled across DIGEST grades 0 and 1 (functional vs. mild impairment) and had a fivefold and sixfold increase for grades 2 and 3 (moderate and severe impairment). Similar to the FOIS–DIGEST grade pair‐wise results, the EAT‐10 differed across all DIGEST grades except between 0 versus 1 and 2 versus 3. This finding is not entirely surprising, as what a patient consumes daily greatly influences their perception of swallowing difficulties. The lack of distinction between DIGEST grades 0 (functional) and 1 (mild) on the EAT‐10 may also relate to the progressive nature and evolution of swallowing impairment in this population and the known compensations individuals living with progressive neurologic disease commonly begin using (many times subconsciously) to adapt to emerging changes and mild impairments in swallowing physiology that mask their awareness and/or reporting of mild swallowing impairment. This finding is also consistent with prior literature on underreporting of dysphagia symptoms in early stages of the disease process [27] and the challenges of self‐reporting dysphagia when the changes are mild or progressive in nature.

Construct validity was evaluated by comparing DIGEST grades with an established ALS bulbar disease severity outcome, namely the ALSFRS‐R bulbar subscale. Similar to other outcomes, ALSFRS‐bulbar subscale scores demonstrated a severity‐dependent decrease (worsening) with increasing DIGEST grades, with DIGEST grades noted to map to this established ALS severity rating scale based on the severity of disease progression. In likeness to the MBSIMP outcomes, ALSFRS‐R bulbar subscale scores were noted to differ between each DIGEST grade comparison except between moderate and severe grades (i.e., 2 vs. 3). The ALSFRS‐R bulbar subscale comprises 3 questions that assess speech, salivation, and swallowing. The single question on swallowing rated on a 4‐point scale is primarily based on oral intake, with only one item relating to perceived swallowing difficulty (*4—Normal eating habits*, *3—Early eating problems*; *occasional choking*, *2—Dietary consistency changes*, *1—Needs supplemental tube feeding*, *0—NPO* (*exclusively parenteral or enteral feeding*)). It is likely that both levels of severity lend to a combination of dietary changes and/or supplemental tube feeding and, in a clinical setting, may be influenced by an individual's goals for full oral intake continuance on a heavily modified diet versus reducing oral intake and partaking in a variety of textures while utilizing supplemental tube feedings. The decision to proceed toward supplemental tube feeding is often not directly mapped to the severity of dysfunction and is made following a shared decision‐making meeting between the individual and the health‐care provider. This variability may explain the lack of discriminant variability between moderate and severe.

Although this study included a relatively large number of pALS with a comprehensive set of accompanying clinical data, only one participant had a DIGEST grade of 4 (life‐threatening dysphagia). This is likely an inevitable issue with any future study, given that to ethically enroll participants in swallowing research studies, an inclusion criterion is that they are still consuming some form of oral intake; however, this does represent a limitation and an area for future study and improvement. Larger datasets may also be required to further examine the ability of DIGEST to discriminate between moderate and severe dysphagia in pALS, given small numbers for subset analysis in the present dataset.

## 5 Conclusion

In this study, the DIGEST scale demonstrated excellent intra‐ and inter‐rater reliability and validity to index swallowing impairment in pALS. Overall, DIGEST grades were able to distinguish between normal and impaired swallowing physiology, oral intake, and patient‐perceived swallowing impairment. Further, the DIGEST scale was able to distinguish between mild versus moderate and severe pharyngeal dysphagia; however, it did not differentiate moderate and severe dysphagia severity levels based on swallowing physiology or ALS severity to distinguish between these more advanced severity levels. These findings provide psychometric validation for the use of the DIGEST scale in pALS and support the clinical utility of this instrument as a global metric of pharyngeal swallowing function in pALS.

## Author Contributions

**Veena Kallambettu:** data analyses, writing‐original draft preparation. **Justine Dallal York:** data collection, data analyses. **Terrie Vasilopolous:** statistical design. **Katherine Hutcheson:** writing/critical review. **Emily Plowman:** design research study, data collection, data analyses, writing, critical review, supervision.All authors have read and approved the final manuscript.

## Conflicts of Interest

The authors declare no conflicts of interest.

## Data Availability

The data that support the findings of this study are available from the corresponding author upon reasonable request.
